# Method and key points for isolation of human amniotic epithelial cells with high yield, viability and purity

**DOI:** 10.1186/s13104-017-2880-6

**Published:** 2017-11-02

**Authors:** Hossein Motedayyen, Nafiseh Esmaeil, Nader Tajik, Fahimeh Khadem, Somayeh Ghotloo, Behnaz Khani, Abbas Rezaei

**Affiliations:** 1 Department of Immunology, Faculty of Medicine, Isfahan University of Medical Sciences, Hezar-Jereeb Ave, Isfahan, Iran; 2 Immunology Research Center (IRC), Iran University of Medical Sciences, Tehran, Iran; 3 Department of Laboratory Medicine, Kashan University of Medical Sciences, Kashan, Iran; 4 Department of Gynecology and Obstetrics, Al-Zahra Hospital, Isfahan University of Medical Sciences, Isfahan, Iran

**Keywords:** hAECs, Placenta, Amnion, Epithelial cells, Isolation

## Abstract

**Objective:**

Human amniotic epithelial cells (hAECs) which are isolated from the amniotic membrane have stem cell-like properties and immunomodulatory effects. Several protocols have been proposed for isolation of hAECs, nevertheless, there is no report concerning isolation of highly viable hAECs, with desirable yield, and without significant purity reduction. In the current study, a detailed protocol with some modification of previous ones is presented in which the amendments led to isolation of hAECs with high purity, yield and viability. Moreover, isolated hAECs were subjected to immuno-phenotyping and their physiological status was assessed using a proliferation assay.

**Results:**

The average yield of obtained hAECs using the new modified method was 190 × 10^6^ cells with a mean viability of 87%, with less than 1% contamination with mesenchymal stem cells (MSCs). The isolated cells were > 95% positive for the epithelial cell markers. The lowest initial plating efficiency of the cells was 80%. Freshly isolated hAECs had the ability to proliferate for 5–6 passages in a standard culture medium.

## Introduction

Human placenta consists of three layers including amnion, chorion and deciduas [1]. The amnion layer, which is derived from the embryo, is the closest layer to the fetus and consists of both cubical and columnar epithelial cells [2–4]. These human amniotic epithelial cells (hAECs) have unique properties that distinguish them from other human cell types including immunomodulatory effects and stem cell-like features which provide capability to differentiate into different cell types originating from three germ layers without any of the ethical concerns related to human stem cells [1, 5–8]. In addition, no tumorigenicity and transplant rejection were observed upon hAECs transplantation [1, 9]. Therefore, hAECs could be safely employed in regenerative medicine and treatment of diseases with immune pathophysiology.

Until now, several protocols were reported by which hAECs were isolated with the average yield ranging from 8 × 10^6^ to 120 × 10^6^ cells and a viability of 83–98%. The isolated cells were positive for the epithelial cell markers in range of 83–98%, while contamination with MSCs was among 1–80% [3, 10–15], nevertheless, as mentioned in Table 1, no report is available concerning isolation of highly viable hAECs with desirable yield and without any significant purity reduction. In this study, a detailed protocol with some modification of previous ones is presented in which by using the new modified method hAECs were isolated with high yield, viability and purity with the minimum contamination with other cell populations. In addition, the isolated hAECs were subjected to immuno-phenotyping, and their physiological status was assessed using a proliferation assay.

**Table 1:** Viability, yield and purity of isolated hAECs using different protocols

Article name	Authors	Published year	Viability (%)	Yield (per placenta)	Percentage of cells with epithelial cell marker	Percentage of cells with stem cell markers
Isolation of human amnion epithelial cells according to current good manufacturing procedures	Roberto Gramignoli et al.	2016	94.4 ± 0.3%	80–300 × 10^6^ (> 107 cells/g of processed tissue)	> 85% CD49f and EpCAM positive cells	< 15% CD105 positive cells
Isolation, cryopreservation and culture of human amnion epithelial cells for clinical applications	Sean V. Murphy et al.	2014	83 ± 4%	120 ± 40 × 10^6^	92% Ep-CAM positive cells	< 1% CD90 & CD105 positive cells
Isolation and Partial characterization of human amniotic epithelial cells: the effect of trypsin	Meraj Tabatabaei et al.	2014	> 98%	80–130 × 10^6^	> 99% cytokeratin positive cells	> 56% CD105 positive cells
Amnion epithelial cell isolation and characterization for clinical use	Sean Murphy et al.	2010	83 ± 4%	120 ± 40 × 10^6^	92% Ep-CAM positive cells	< 1% CD90 & CD105 positive cells
Isolation of amniotic epithelial stem cells	Toshio Miki et al.	2010	Not reported	80–300 × 10^6^	Not reported	8.79 ± 2.84% SSEA-3 positive cells, 43.94 ± 14.8% SSEA-4 positive cells, 9.82 ± 4.31% TRA 1-60 positive cells & 9.91 ± 4.49% TRA 1-81 positive cells
Human amniotic epithelial cells: isolation and characterization	Ruth Gomez Dominguez	2008	Not reported	12 × 10^6^	97% cytokeratin 19 positive cells	30% CD105 positive cells
The potential of amniotic membrane/amnion-derived cells for regeneration of various tissues	Ayaka Toda et al.	2007	Not reported	8–50 × 10^6^	Not reported	Not reported

## Main text

### Note

All deliveries which were positive for infectious agents including HBV, HCV and HIV and those with pre-diagnosed genetic abnormalities were excluded from this study. Full-term human placentas were obtained from six healthy women with a normal singleton pregnancy undergoing uncomplicated elective cesarean section. All the materials and equipment used in this procedure were sterile.

### Methods

#### Preparation of reagents and solutions

##### Standard culture medium

DMEM/F12 (Gibco, Thermo Fisher Scientific, USA) supplemented with 10% FBS, 100 U/ml penicillin, 100 μg/ml streptomycin (1X, Sigma Aldrich, USA) and 10 ng/ml EGF (Thermo Fisher Scientific, USA) was used as standard culture medium.

##### Pre-digestion buffer

To prepare 1000 ml of 1X Pre-digestion buffer, all the components mentioned in Table 2 were added to 900 ml of tissue culture-grade water (20–25 °C) and the pH was adjusted to 7.2 (It is because pH of the buffer rises up to 0.1–0.3 units during filtration). Thereafter, tissue culture-grade water was added to the solution and the final volume adjusted to 1000 ml. The buffer was immediately sterilized by filtration using a 0.22 µm filter (Thermo Fisher Scientific, USA) and stored at 4 °C for further usage.

**Table 2:** Pre-digestion buffer ingredients

	gr/litter
Sodium chloride (NaCl)	8
Potassium chloride (KCL)	0.4
Sodium bicarbonate (NaHCO_3_)	0.35
D-glucose (Dextrose)	1
Potassium diHydrogen phosphate (KH_2_PO_4_)	0.06
Sodium EDTA (Na2-EDTA)	0.2
Sodium hydrogen phosphate, anhydrous (Na_2_HPO_4_)	0.047

### Isolation of hAECs from placenta

#### Isolation of the amniotic membrane

**Fig. 1:**
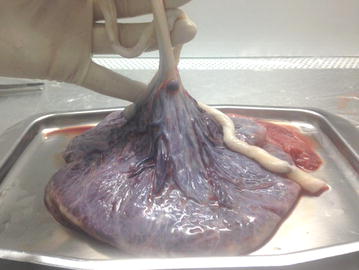
The position of placing placenta in a sterile container in which the fetal surface was faced up

**Fig. 2:**
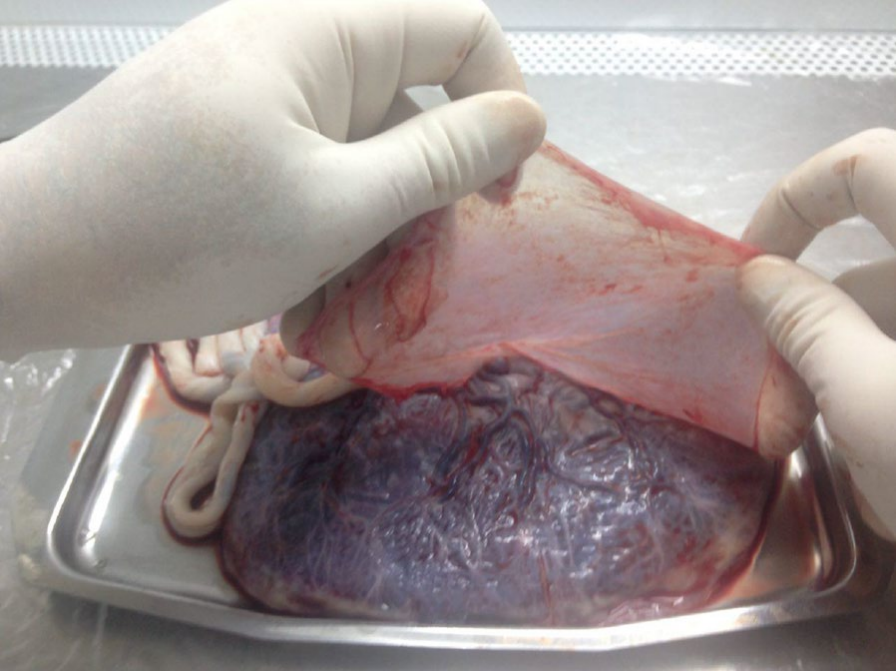
Manual stripping of the amniotic membrane (upper layer) of the placenta from the chorion layer

**Fig. 3:**
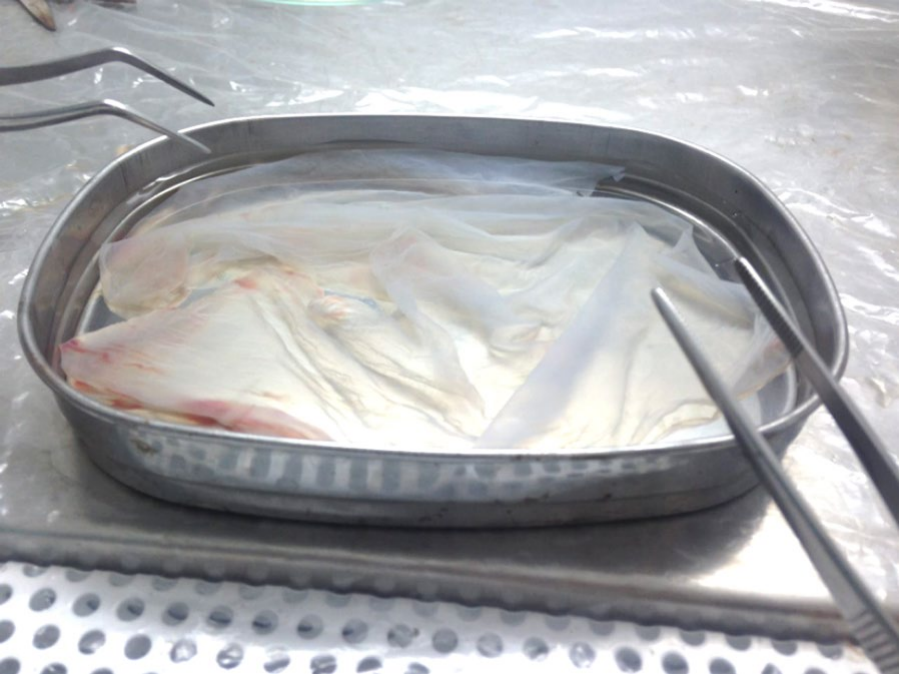
Washing the isolated amnion membrane with ice-cold PBS

The placenta was transferred to the laboratory in a sterile container which was filled with ice-cold Hanks’ balanced salt solution (calcium- and magnesium-free HBSS) or RPMI/1640 (Gibco, Thermo Fisher Scientific, USA) containing 1% penicillin/streptomycin solution (Sigma Aldrich, USA) to cover up the placenta surface. The placenta was placed in a sterile container (under a horizontal laminar flow hood) while the amniotic membrane was faced up (Fig. 1). The amnion membrane was manually stripped from the chorion layer, starting from the outer edge of the amniotic membrane and continuing towards the umbilical cord (Fig. 2). The membrane was washed several times with ice-clod PBS (pH 7.2) to remove blood clots, torn pieces and cellular debris (Fig. 3).

#### Digestion of the amniotic membrane

Blood-free amnion was cut into pieces around 7 cm long and transferred with forceps to two new 50 ml falcon conical tubes. To each tube 20 ml pre-digestion buffer was added, and then incubated at 37 °C for 15 min with gentle shaking (30–60 RPM). Afterwards, the amnion pieces were transferred into three new 50 ml falcon conical tubes containing 20 ml of pre-warm 0.05% trypsin/EDTA (Thermo Fisher Scientific, USA) and incubated at 37 °C for 10 min with gentle shaking (first digestion). The obtained cells at this step were discarded to exclude blood clots and cellular debris and the membrane pieces were transferred into new tubes. The enzymatic digestion was followed by addition of 20 ml pre-warm 0.05% trypsin/EDTA and incubation at 37 °C for 30 min with gentle shaking (second digestion). This step was repeated, then the second and the third digestions were neutralized for trypsin activity by adding 30 ml HBSS, and finally centrifuged at 200×*g* for 5 min at 4 °C. The cell pellets were re-suspended in 10 ml standard culture medium, mixed together and filtered through a 100 μm cell strainer (Thermo Fisher Scientific, USA). The filtrate cell number was counted using a haemocytometer and cell viability was determined using trypan blue dye exclusion.

### Key points

1. It is possible that the amnion layer is peeled off from the chorion in the operating room, which has its advantages including: using less transportation medium, minimize possibility of non-sterile samples, and decreasing the amnion contamination with blood clots.
2. It is recommended that before hAECs isolation, a piece of the amniotic membrane be observed under an optical microscope (40 × magnification) in order to check the status of the cells. An amniotic membrane with epithelial cells, which are notably vacuolated in their cytoplasm, is not suitable for cell isolation (Fig. 4A, B). Do not process to the next steps.

**Fig. 4:**
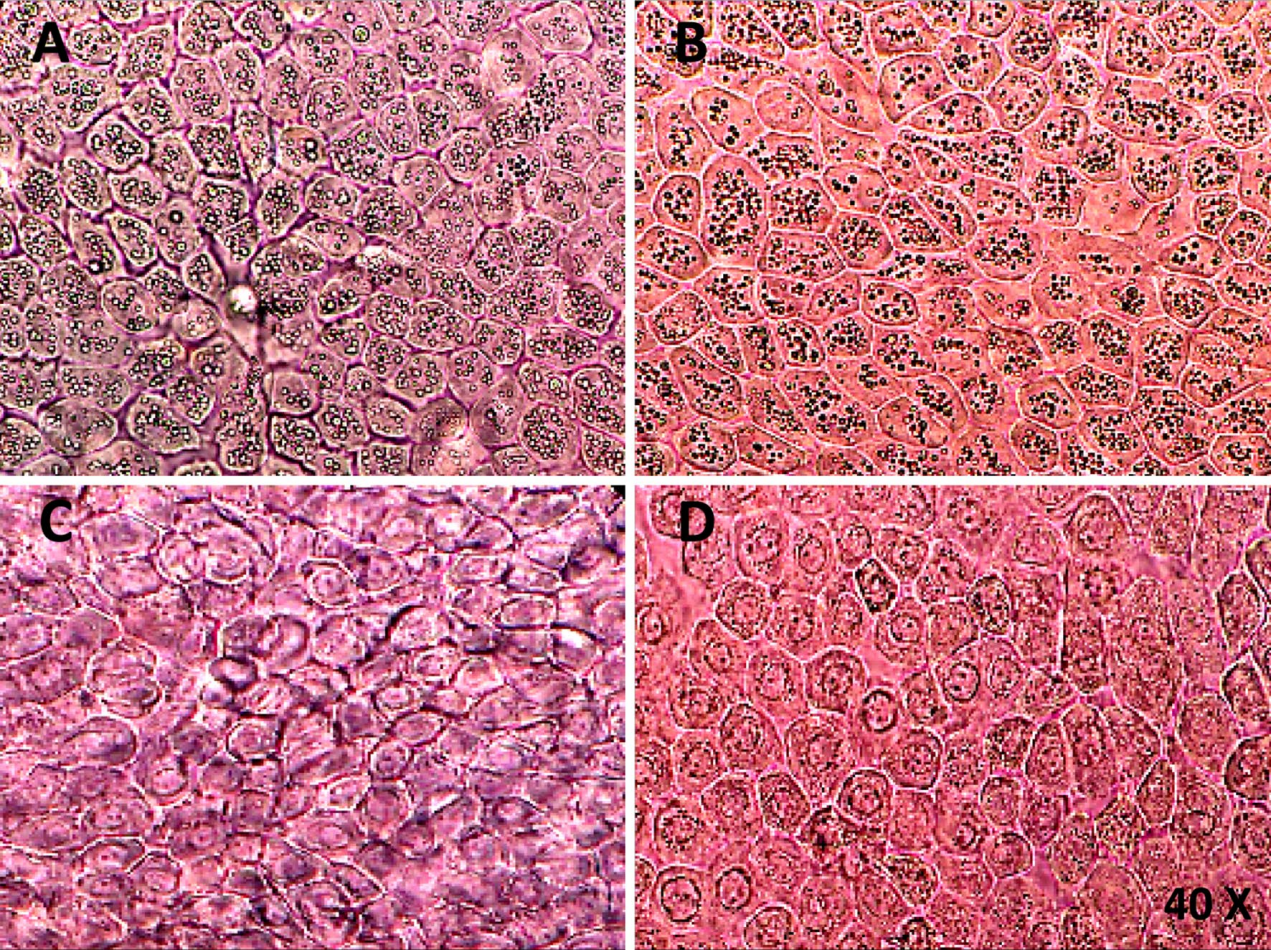
Epithelial cells of the amniotic membrane under an optical microscope. **A**, **B** An amniotic membrane with epithelial cells which are fully vacuolated in their cytoplasm. **C**, **D** An amniotic membrane with normal epithelial cells which is suitable for hAECs isolation
3. It is suggested that the status of the remained cells on the amniotic membrane after each digestion step be checked under an optical microscope and the membrane with highly vacuolated cells is discarded. Checking a piece of the amniotic membrane under a microscope after each digestion step also helps to realize whether the next-step of enzymatic digestion is required or not. Accordingly, the number of required digestion steps to separate majority of the cells from the membrane may be increased or decreased.
4. It is suggested that at the end of each digestion, the membrane pieces be gently shacked using a forceps in 50 ml tubes containing the trypsin digest to separate all the epithelial cells in case still be (loosely) attached to the membrane.

### hAECs immuno-phenotyping using flow cytometry

The purity and phenotypic characteristics of freshly isolated hAECs were determined using flow cytometry. The cells (4–8 × 10^5^) were stained with different antibodies (Table 3) or matched-isotype control IgG at 4 °C for 25 min. Matched isotype control antibodies were used as negative controls and MSCs were employed as positive control for anti-CD90 and anti-CD105. Afterwards, the cells were washed three times using cell staining buffer (Biolegend, USA) and centrifugation at 200×*g* for 5 min at 4 °C. Intracellular staining with FITC-conjugated–anti-cytokeratin (Biolegend, USA) was performed after fixation and cells permeabilization according to the manufacturer’s instructions (eBioscience, USA). The data was acquired using a FACSCalibur system (Becton–Dickinson, CA) and analyzed using CellQuest software (Becton–Dickinson, CA).

**Table 3:** Used antibodies to determine phenotypic characterictics of hAECs by flow cytometry

Primary antibodies/ fluorochrome	Isotype	Catalog number	Source of primary antibodies
Alexa Fluor® 488 anti-Cytokeratin (pan reactive)	Mouse IgG1, κ (cat. no:400143)	628608	Biolegend, San Diego, CA, USA
FITC anti-human CD105	Mouse IgG1, κ (cat. no:400107)	323203	Biolegend, San Diego, CA, USA
FITC anti-human CD90	Mouse IgG1, κ (cat. no:400107)	328107	Biolegend, San Diego, CA, USA
FITC anti-human CD45	Mouse IgG1, κ (cat. no:400107)	368507	Biolegend, San Diego, CA, USA
FITC anti-human CD14	Mouse IgG1, κ (cat. no:400107)	367115	Biolegend, San Diego, CA, USA
FITC anti-human CD4	Mouse IgG1, κ (cat. no:400107)	357405	Biolegend, San Diego, CA, USA
FITC anti-human CD8a	Mouse IgG1, κ (cat. no:400107)	300905	Biolegend, San Diego, CA, USA
PE anti-human CD56	Mouse IgG1, κ (cat. no:400111)	355503	Biolegend, San Diego, CA, USA
FITC anti-human CD3	Mouse IgG1, κ (cat. no:400107)	362305	Biolegend, San Diego, CA, USA
FITC mouse anti-human HLA-DR	Mouse IgG_2a_, κ(cat. no: 555057)	555560	BD Biosciences, San Jose, CA, USA
FITC mouse anti-human CD34	Mouse IgG_1_, κ(cat. no: 555748)	555821	BD Biosciences, San Jose, CA, USA
FITC mouse anti-human CD38	Mouse IgG_1_, κ(cat. no: 555748)	555459	BD Biosciences, San Jose, CA, USA
PE mouse anti-human CD44	Mouse IgG_1_, κ(cat. no: 550617)	550989	BD Biosciences, San Jose, CA, USA
PE mouse anti-human CD9	Mouse IgG_1_, κ(cat. no: 550617)	555372	BD Biosciences, San Jose, CA, USA
PE mouse anti-human CD29	Mouse IgG_1_, κ(cat. no: 550617)	557332	BD Biosciences, San Jose, CA, USA
PE mouse anti-human CD73	Mouse IgG_1_, κ(cat. no: 550617)	550257	BD Biosciences, San Jose, CA, USA
Anti-human SSAE-4 PE	Mouse/ IgG3(cat. no:12-4742-42)	12-8843-42	Thermo Fisher Scientific, Waltham, MA, USA
Anti-human CD133 FITC	Mouse/ IgG2b, kappa(cat. no: 11-4732-42)	11-1339-42	Thermo Fisher Scientific, Waltham, MA, USA

### hAECs proliferation assay

Isolated hAECs were cultured in 75 cm^2^ tissue culture flasks at a density of 2.5 × 10^5^ cells/cm^2^ using standard culture medium, and then incubated at 37 °C with 5% CO_2_. The initial plating efficiency of the cells was determined after 2 days of incubation. The hAECs were dissociated using 0.05% trypsin–EDTA solution, whenever they were approximately 80% confluent. The cells were sub-cultured in 1:4 ratios in standard culture medium.

## Results

### The yield and viability of hAECs

**Fig. 5:**
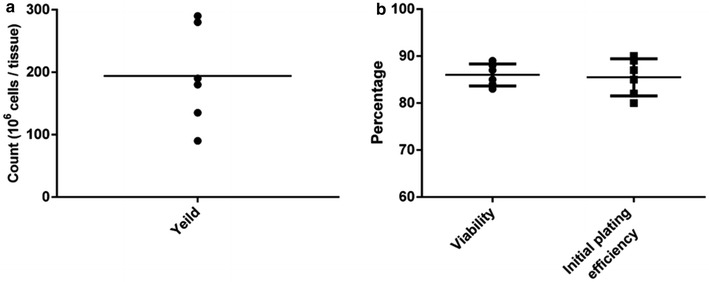
Yield and viability of isolated hAECs. **a** The average yield of isolated hAECs was 190 × 10^6^ cells with a typical range of 90–280 million cells. **b** An average viability obtained using this protocol was 87% (ranging from 83 to 89%). The depicted results are representative of six independent experiments

The average yield of obtained hAECs by this method was 190 × 10^6^ cells with a typical range of 90–280 million cells (Fig. 5a). Noticeably, for this yield the average obtained viability was 87% (ranging from 83 to 89%) (Fig. 5b).

### Immuno-phenotyping of hAECs

**Fig. 6:**
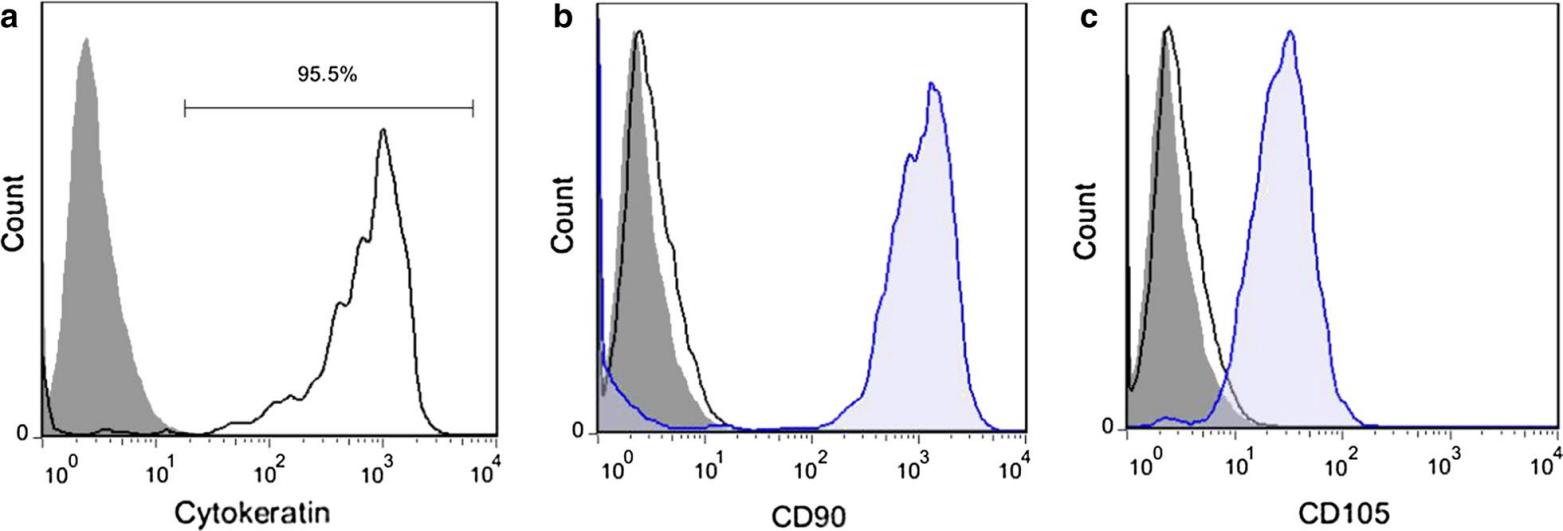
The purity of isolated hAECs. **a** More than 95% of the isolated hAECs were positive for cytokeratin, the epithelial cell marker. **b**, **c** Less than 1% of isolated hAECs were positive for MSC markers CD90 (**b**) and CD105 (**c**). Gray shaded histogram: hAECs were stained with matched isotype control antibodies as negative controls (**a**–**c**). Blue shaded histogram: MSCs were stained with anti-CD90 (**b**) and anti-CD105 (**c**) antibodies as positive controls. Black line: hAECs were stained with anti-cytokeratin (**a**), anti-CD90 (**b**) and anti-CD105 (**c**). The indicated results are representative of six independent experiments

The hAECs purity achieved by this protocol was at least 95.42%, as confirmed by cytokeratin analysis, an epithelial cell marker (Fig. 6a). Less than 1% of the isolated cells were positive for MSC markers (CD90, CD105) (Fig. 6b, c). In addition, hAECs immuno-phenotyping from different donors showed that the isolated cells were almost a homogenous population (Table 4).

**Table 4:** Determination of markers of hAECs by flow cytometry

Marker	Donor1	Donor2	Donor3	Donor4	Donor5	Donor6
Pan cytokeratin	++++	++++	++++	**++++**	**++++**	**++++**
CD73	+++	+++	+++	**+++**	**+++**	**+++**
SSEA-4	+++	+++	+++	**+++**	**+++**	**+++**
CD133	-	-	-	**-**	**-**	**-**
CD90	-	-	-	**-**	**-**	**-**
CD105	-	-	-	**-**	**-**	**-**
CD34	-	-	-	**-**	**-**	**-**
CD45	-	-	-	**-**	**-**	**-**
CD9	++	-	+	**+**	**-**	**+**
CD38	-	-	-	**-**	**-**	**-**
HLA-DR	-	-	-	**-**	**-**	**-**
Integrin-β2 (CD29)	+	+	+	**+**	**++**	**+**
Hyaluronic acid receptor (CD44)	-	+	+	**+**	**++**	**+**
CD56	-/+	-/+	-/+	**-/+**	**-/+**	**-/+**
CD14	+	++	-	**+**	**++**	**-**
CD3	-	-	-	**-**	**-**	**-**
CD4	-	-	-	**-**	**-**	**-**
CD8	-	-	-	**-**	**-**	**-**

–, Not determined; ∓, very low expression (< 10%); +, low expression (10–30%); ++, intermediate expression (30–60%); +++, high expression (60–90%); ++++, very high expression (> 90%)

### The proliferation capability of hAECs

After 48 h, the initial plating efficiency of the isolated hAECs on culture dishes was at least 80% (Fig. 5b). Freshly isolated hAECs had the ability to proliferate for 5–6 passages in standard culture medium. In contrast with some reports [5, 16], hAECs which were cultured without EGF were not able to proliferate at all.

## Discussion

hAECs are a type of stem cells isolated from the amniotic membrane of the placenta. In addition to stem cell-like properties, which proposed hAECs as a potential candidate for regenerative medicine, it has been shown that they can be used as an immunomodulatory agent in treatment of diseases with immune pathophysiology.

hAECs isolation from the amniotic membrane is performed with enzymatic digestion of the amniotic membrane, since amnion epithelium is a single monolayer with a weak cell–cell adhesion at the lateral sides and trypsin is able to separate hAECs from the amniotic basement membrane [11]. There is a notable variability in the purity, yield and viability of hAECs isolation using previous protocols [3, 10–15]. These variations might be related to several factors including mother related parameters (e.g. age, gestational week and delivery type), size and quality of the placenta (which is affected by duration of the time between the delivery and hAECs isolation), transportation condition (such as cold chain), residual blood on the tissue (in spite of extensive washing), type and concentration of the enzyme solution, and enzymatic digestion time [3, 10–14]. A disadvantage of these methods for cell isolation is that achieving a desirable yield come at the expense of losing the cell viability and purity. The presented protocol at this study resulted in hAECs isolation with high yield (to our knowledge, the highest yield reported yet), viability and purity.

Using the proposed modified protocol, an average yield of 190 × 10^6^ hAECs (in range of 90–280 million cells) was achieved compared to the average yield of 8 × 10^6^–120 × 10^6^ cells obtained using previous protocols [3, 10–12, 14, 15]. Regarding the fact that a consistent protocol was followed for hAECs isolation from all donors in the current study, the observed extensive variability in cell yield might be related to quality of the placenta obtained from different donors. The mean viability of isolated hAECs in this study was 87%, compared to previously reports with a range from 83 to 99% [14]. Previous methods reported that high yield of the viable cells was accompanied contamination with other cell populations [10, 11, 14]. However, the current study reported a highly viable hAECs at high yield and minimum contamination with MSCs. These findings could be mainly attributed to two factors, a shorter incubation time compared to previous protocols and discarding the first digestion. Regarding the facts that a part of blood clots and cellular debris are not removed after extensive washing of the amniotic membrane with PBS, consequently these parts are separated from the membrane in the first step of digestion.

The initial plating efficiency of the cells in the current study was at least 80% after 48 h, moreover the cells continued to proliferate for 5–6 passages in standard culture medium. It is found that in addition to the initial viability of the isolated cells which is an essential factor for determining long-term survival of hAECs and their proliferation, the initial plating efficiency is critical as well.

According to the results, isolated hAECs from different placentas were highly homogeneous based on the expression of epithelial (cytokeratin), MSC (CD105, CD73, CD90), embryonic stem cell (SSEA-4), hematopoietic stem cell (CD34), and immunologic (HLA-DR, CD56, CD3, CD4, CD8) markers and adhesion molecules (CD29). However, there were differences in the level of CD9, CD14 and CD44 between different donors.

In summary, using the new modified protocol a high hAECs yield with high viability and purity was obtained, and the cells kept their proliferation ability until passage 5–6.

## Limitation

A limitation of this technique is that increasing the cells yield may result in losing the viability, purity and initial plating efficiency.

## Abbreviations

DMEM: Dulbecco’s modified eagle’s medium
RPMI: Roswell park memorial institute
PBS: phosphate-buffered saline
FBS: fetal bovine serum
EGF: epidermal growth factor
hAECs: human amniotic epithelial cells
HBSS: Hanks’ balanced salt solution
HBV: hepatitis B virus
HCV: hepatitis C virus
HIV: human immunodeficiency virus
